# Predictive Biomarkers for the Ranking of Pulmonary Toxicity of Nanomaterials

**DOI:** 10.3390/nano10102032

**Published:** 2020-10-15

**Authors:** Chinatsu Nishida, Hiroto Izumi, Taisuke Tomonaga, Jun-ichi Takeshita, Ke-Yong Wang, Kei Yamasaki, Kazuhiro Yatera, Yasuo Morimoto

**Affiliations:** 1 Department of Respiratory Medicine, University of Occupational and Environmental Health, Japan. 1-1 Iseigaoka, Yahata-nishi-ku, Kitakyushu, Fukuoka 807-8555, Japan; c-nishi@med.uoeh-u.ac.jp (C.N.); yamasaki@med.uoeh-u.ac.jp (K.Y.); yatera@med.uoeh-u.ac.jp (K.Y.); 2 Department of Occupational Pneumology, Institute of Industrial Ecological Sciences, University of Occupational and Environmental Health, Japan. 1-1 Iseigaoka, Yahata-nishi-ku, Kitakyushu, Fukuoka 807-8555, Japan; h-izumi@med.uoeh-u.ac.jp (H.I.); t-tomonaga@med.uoeh-u.ac.jp (T.T.); 3 Research Institute of Science for Safety and Sustainability, National Institute of Advanced Industrial Science and Technology (AIST), Tsukuba, Japan. 16-1 Onogawa, Tsukuba, Ibaraki 305-8569, Japan; jun-takeshita@aist.go.jp; 4 Shared-Use Research Center, University of Occupational and Environmental Health, Japan. 1-1 Iseigaoka, Yahata-nishi-ku, Kitakyushu, Fukuoka 807-8555, Japan; kywang@med.uoeh-u.ac.jp

**Keywords:** biomarker, pulmonary toxicity, chemokine, nanomaterials

## Abstract

We analyzed the mRNA expression of chemokines in rat lungs following intratracheal instillation of nanomaterials in order to find useful predictive markers of the pulmonary toxicity of nanomaterials. Nickel oxide (NiO) and cerium dioxide (CeO_2_) as nanomaterials with high pulmonary toxicity, and titanium dioxide (TiO_2_) and zinc oxide (ZnO) as nanomaterials with low pulmonary toxicity, were administered into rat lungs (0.8 or 4 mg/kg BW). *C-X-C motif chemokine 5* (*CXCL5*), *C-C motif chemokine 2* (*CCL2*), *C-C motif chemokine 7* (*CCL7*), *C-X-C motif chemokine 10* (*CXCL10*), and *C-X-C motif chemokine 11* (*CXCL11*) were selected using cDNA microarray analysis at one month after instillation of NiO in the high dose group. The mRNA expression of these five genes were evaluated while using real-time quantitative polymerase chain reaction (RT-qPCR) from three days to six months after intratracheal instillation. The receiver operating characteristic (ROC) results showed a considerable relationship between the pulmonary toxicity ranking of nanomaterials and the expression of *CXCL5*, *CCL2*, and *CCL7* at one week and one month. The expression levels of these three genes also moderately or strongly correlated with inflammation in the lung tissues. Three chemokine genes can be useful as predictive biomarkers for the ranking of the pulmonary toxicity of nanomaterials.

## 1. Introduction

Nanoparticles are defined as particles with at least one dimension of 100 nm or less [1,2] and, in recent years, the demand for industrial nanomaterials composed of these nanoparticles has increased significantly. New industrial nanomaterials are being created one after another by nanotechnology for controlling functions on the nanometer scale, and they are being used for various applications in various fields. The pulmonary toxicity of nanomaterials needs to be fully assessed before the industrial nanomaterials are handled by humans. It is necessary to develop biomarkers in order to predict the hazard level of industrial nanomaterials, because multi-wall carbon nanotubes (MWCNTs), a representative nanomaterial, are known to be carcinogenic: the development of lung tumors or malignant pleural mesothelioma was confirmed in intratracheal instillation [3,4] and in inhalation studies [5].

In pulmonary disorders that are caused by respirable chemicals, it is considered that the chemical deposits in the lungs cause sustained inflammation and ultimately the formation of chronic and irreversible lesions, such as lung fibrosis and tumors [6,7,8,9,10]. It has been reported, for example, that asbestos and crystalline silica, which have high pulmonary toxicity, cause sustained inflammation in the lungs, leading to irreversible fibrosis, lung cancer and mesothelioma [11,12]. Thus, sustained inflammation is considered to be an important process in the induction of chronic and irreversible lesions of the lung [6,7,8,9,10,13]. MWCNTs, which are carcinogenic, have shown sustained inflammation in the lung following inhalation and intratracheal instillation [14,15,16,17]. Taken together, it is thought that ‘sustained inflammation’ is an important process in predicting lung disorders that are caused by industrial nanomaterials. It is considered that the detection of biomarkers that reflect sustained inflammation in the lung can lead to early detection of the hazardous effects of nanomaterials and, thus, can predict the progression to chronic/irreversible lesions. Sustained inflammation is composed of neutrophils and alveolar macrophages, and its pathogenesis is thought to be associated with cytokines, especially chemokines [6,9,18].

In this study, we focused on inflammation-related genes that are based on the results of a comprehensive gene expression analysis using cDNA microarray, and examined whether or not biomarkers for predicting lung disorder by nanomaterials can be detected following the intratracheal instillation of nanomaterials with different pulmonary toxicities.

## 2. Materials and Methods

### 2.1. Sample Nanomaterials

We used nickel oxide (NiO), cerium dioxide (CeO_2_), titanium dioxide (TiO_2_), and zinc oxide (ZnO) as industrial nanomaterials in the present study. Commercially available NiO (US3355, US Research Nanomaterials, Houston, TX, USA), CeO_2_ (Wako Chemical, Ltd., Osaka, Japan), TiO_2_ (Rutile) (MT-150AW, Teyca Co. Ltd., Osaka, Japan), and ZnO (Sigma-Aldrich Co. LLC., Tokyo, Japan) were dispersed in 0.4 mL distilled water. Table 1 shows the physicochemical profiles of these samples [19,20,21,22,23,24]. Transmission electron microscope (TEM) images of each four nanoparticle suspension are shown in our previous reports [20,21,24]. We defined the toxicity of the chemicals, as follows: the chemicals that induced either sustained inflammation, fibrosis, or tumors were set as having high pulmonary toxicity, and the chemicals that did not induce any of those pathological lesions were set as having low pulmonary toxicity. Accordingly, NiO and CeO_2_ were classified as nanomaterials with high pulmonary toxicity [10,19,20,25,26], and TiO_2_ and ZnO were classified as nanomaterials with low pulmonary toxicity [19,21,27,28,29].

### 2.2. Animals

Male Fischer 344 rats (9–11 weeks old) that were used for exposure to nanomaterials were purchased from Charles River Laboratories International, Inc., Kanagawa, Japan. The animals were kept in the Laboratory Animal Research Center of the University of Occupational and Environmental Health for two weeks with free access to a commercial diet and water. All of the procedures and animal handling were done according to the guidelines that were described in the Japanese Guide for the Care and Use of Laboratory Animals were approved by the Animal Care and Use Committee, University of Occupational and Environmental Health, Japan (animal studies ethics clearance proposal number; AE11-012).

### 2.3. Intratracheal Instillation

The NiO, CeO_2_, TiO_2_, and ZnO nanomaterials were suspended in 0.4 mL distilled water. Doses of 0.2 mg (low dose, equivalent to 0.8 mg/kg BW) or 1 mg (high dose, equivalent to 4 mg/kg BW) were administered to rats (12 weeks old) in a single intratracheal instillation. Each of the negative control groups received distilled water.

### 2.4. Animals Following Intratracheal Instillation

In the exposure to the four different nanomaterials and the negative control, there were five rats in each group at each time point. The animals were dissected at three days, one week, one month, three months, and six months after intratracheal instillation and the lung was divided into right and left lungs. Analysis of cDNA microarray and qRT-PCR was performed with the homogenized third lobe of the right lung, and histopathological evaluation was performed with the left lung inflated and fixed by 4% paraformaldehyde or 10% formaldehyde.

### 2.5. Total RNA Extraction

The third lobes of the right lungs (*n* = 5 per group per time point) were homogenized while using a QIAzol lysis reagent with a TissueRupotor (Qiagen, Hilden, Germany). Total RNA from the homogenates was extracted using a miRNeasy Mini Kit (Qiagen, Hilden, Germany) following the manufacturer’s instructions. RNA was quantified while using a NanoDrop 2000 spectrophotometer (Thermo Fisher Scientific Inc., Waltham, MA, USA) and the quality of the samples was analyzed by a Bioanalyzer 2100 (Agilent Technologies, Santa Clara, CA, USA).

### 2.6. Microarray Analysis

We used a three-dimensional (3D)-Gene Rat Oligo Chips 20K (version 1.1) (Toray Industries, Tokyo, Japan), which could mount 20,174 genes, for the DNA microarray analysis. Total RNA extracted from the lungs of the five rats in the NiO-high dose group was mixed in equal amounts to make one sample, and that was amplified by the use of an Amino Allyl MessageAmp II aRNA Amplification Kit (Ambion, Inc., Austin, CA, USA). The negative control group was treated in the same manner. The antisense RNA (aRNA) were labeled with Cy5, using Amersham Cy5 Mono-Reactive Dye (GE Healthcare, Buckinghamshire, UK), and the labeled aRNA were hybridized at 37 °C for 16 h. The hybridization was performed according to the supplier’s protocols [30]. The chips were washed and dried, and then scanned in an ozone-free environment while using a 3D-Gene Scanner 3000 (Toray Industries, Tokyo, Japan) and analyzed by use of 3D-Gene Extraction Software (Toray Industries, Tokyo, Japan). The digitalized fluorescent signals provided by the above-described software were regarded as the raw data. All of the normalized data were globally normalized per microarray, such that the median of the signal intensity was adjusted to 25. The function of the enhanced expression genes was analysed by the Database for Annotation Visualization and Integrated Discovery 6.8 [31].

### 2.7. Validation of Gene Expression Data Using Quantitative Real-Time Polymerase Chain Reaction

qRT-PCR was performed, as described previously [32]. Briefly, the total RNA extracted from the lungs at each observation point in each group were transcribed into cDNA (High-Capacity cDNA^TM^ Kit, Life Technologies, Tokyo, Japan). qRT-PCR assays were performed while using TaqMan (TaqMan Gene Expression Assays, Thermo Fisher Scientific Inc., Waltham, MA, USA) according to the manufacturer’s protocol. Gene expression data were analyzed by the comparative cycle time (ΔΔCT) method while using the 7500 Fast Real-Time PCR System. The Assays-on-Demand TaqMan probes and primer pairs were *CXCL5* (Assay ID Rn00573587_g1), *CCL2* (Assay ID Rn00580555_m1), *CCL7* (Assay ID Rn01467286_m1), *CXCL10* (Assay ID Rn00594648_m1), and *CXCL11* (Assay ID Rn00788261_g1). All of the experiments were performed in a StepOnePlus^TM^ Real-Time PCR Systems (Life Technologies, Tokyo, Japan). All of the expression data were normalized to endogenous control β-actin expression (Assay ID Rn00667869_m1).

### 2.8. Statistical Analysis

Statistical analysis was carried out using JMP^®^ Pro software (JMP Version 14.2.0, SAS Institute Inc., Cary, NC, USA). *p* values < 0.05 were considered to be significant. Dunnett’s tests were used appropriately in order to detect individual differences in the gene expression levels of each of the 5 chemokines between those exposed to the four nanomaterial samples and the negative controls. We assigned the toxicity of the exposure nanomaterials as being high or low according to the gene expression levels of each of the five chemokines of each sample (20 samples for both high and low toxicity at each time point), and analyzed the sensitivity and specificity for high toxicity at each time point to create the receiver operating characteristic (ROC) curves and AUCs. Youden’s Index was used in order to determine the cut-off value. Youden’s Index was defined, as follows: Youden’s Index = sensitivity + specificity-1, where the definitions of sensitivity and specificity are shown in Appendix A Table A1, together with specific examples using a confusion matrix. In the evaluation using the combination of chemokine genes, the cases where the expression of at least one gene was equal to or higher than the cut-off value were defined as positive. Spearman’s rank correlation coefficient was used in order to estimate the correlation between gene expression levels of *CXCL5*, *CCL2*, *CCL7*, *CXCL10*, or *CXCL11* and the score of inflammatory cell infiltration of lung tissue.

### 2.9. Histopathology and Immunohistochemistry

The obtained lung tissue, which was inflated and fixed with 4% paraformaldehyde or 10% formaldehyde under a pressure of 25 cm water, was embedded in paraffin, sectioned at a thickness of 4 μm, and then stained with hematoxylin and eosin (H&E). The slides were assessed for histological changes (H&E stain) by a board-certified pathologist (New Histo. Science Laboratory Co., Ltd., Tokyo, Japan). The severity of the histological changes in the lung in the negative control and nanoparticle-exposed rats was scored as none (0), minimal (0.5), mild (1), moderate (2), or severe (3).

The upregulation of *CXCL5*, *CCL2*, and *CCL7* was evaluated by immunostaining with rabbit anti-mouse CXCL5 polyclonal antibody (1:200 dilution, bs-2549R; Bioss Inc., Woburn, MA, USA), goat anti-rat CCL2 polyclonal antibody (1:200 dilution, sc-1785; Santa Cruz Biotechnologies, Inc., Dallas, CA, USA), and goat anti-mouse CCL7 polyclonal antibody (1:50 dilution, sc-21202; Santa Cruz Biotechnologies, Inc., Dallas, CA, USA), respectively, while using the lung tissue samples from the NiO-high dose group of one month after intratracheal instillation.

## 3. Results

### 3.1. Gene Expression Analysis

**Figure 1:**
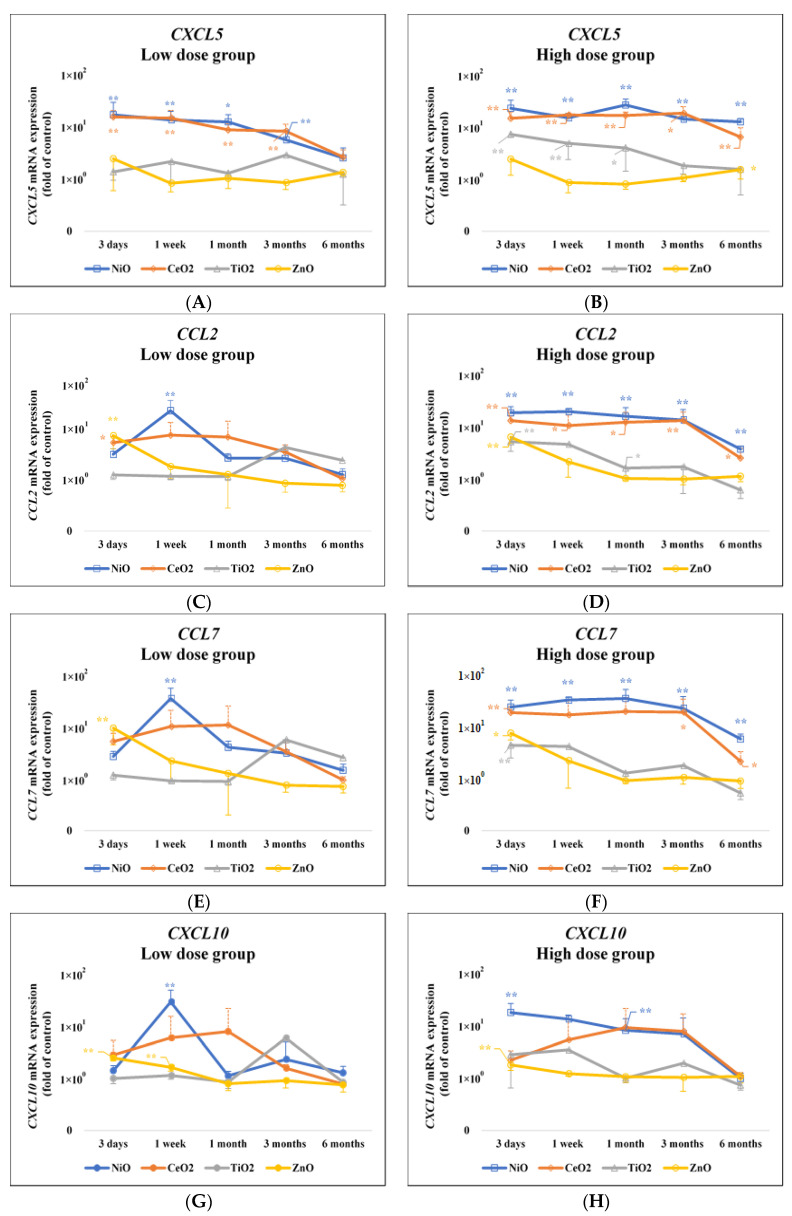

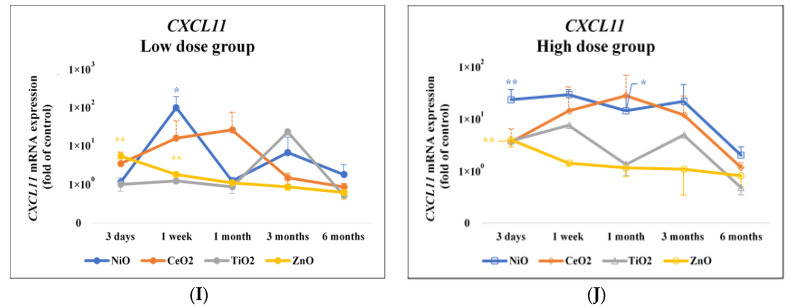
Gene expression of five chemokines in lung exposed to nanomaterials with different pulmonary toxicities. (**A**)*CXCL5* mRNA expression (Low dose group); (**B**) *CXCL5* mRNA expression (High dose group); (**C**) *CCL2* mRNA expression (Low dose group); (**D**) *CCL2* mRNA expression (High dose group); (**E**) *CCL7* mRNA expression (Low dose group); (**F**) *CCL7* mRNA expression (High dose group); (**G**) *CXCL10* mRNA expression (Low dose group); (**H**) *CXCL10* mRNA expression (High dose group); (**I**) *CXCL11* mRNA expression (Low dose group); (**J**) *CXCL11* mRNA expression (High dose group). Data, normalized to β-actin endogenous control, are presented as fold change relative to the negative controls (distilled water). Values changes are mean ± standard deviation (SD) (*p* < 0.05, *n* = 5). Increased expression of *CXCL5* gene in the NiO and CeO_2_ groups was persistently higher, and that in the TiO_2_ and ZnO groups transiently higher compared with the negative control groups, respectively. *CCL2* and *CCL7* also showed a similar tendency to *CXCL5* (* *p* < 0.05, ** *p* < 0.01). The low dose groups: 0.2 mg; the high dose groups: 1.0 mg. Value of approximate 1 × 10^0^ means the negative control.

Table 2 shows the results of gene expression in the NiO-high dose group by cDNA microarray at one month after intratracheal instillation. The number of genes whose expression was increased eight times or more was 16 (Table 2A), and 5 genes among them were chemokine genes (*C-X-C motif chemokine 5* (*CXCL5*)*, C-C motif chemokine 2* (*CCL2*)*, C-C motif chemokine 7* (*CCL7*)*, C-X-C motif chemokine 10* (*CXCL10*), and *C-X-C motif chemokine 11* (*CXCL11*)) (Table 2B). Figure 1 and Appendix A Table A2 show the validated expression levels of the five chemokine genes induced by the four nanomaterials following intratracheal instillation using qRT-PCR over the observation time. The gene expression of *CXCL5* in the lung tissue that was exposed to NiO and CeO_2_, which have high pulmonary toxicity, was persistently high as compared with the negative control throughout the observation time, while the expression was increased transiently or not increased in the lung tissue exposed to TiO_2_ and ZnO, which have low pulmonary toxicity, during the observation time. The nanomaterials with high pulmonary toxicity induced persistent expression patterns of the *CXCL5* gene during the observation period, and the nanomaterials with low pulmonary toxicity did not, or had transient patterns during the observation period.

The expression patterns of *CCL2* and *CCL7* also showed a similar tendency to *CXCL5*. *CCL2* genes had a significantly persistent increase in the NiO and CeO_2_ high dose groups, and *CCL7* genes had a significantly persistent increase in the NiO high dose group. Both of the genes had an insignificant increase in the NiO and CeO_2_ low dose groups and in the CeO_2_ high dose group. There was a transient increase in the TiO_2_ and ZnO-exposed groups at three days or one month following intratracheal instillation.

On the other hand, the gene expression of *CXCL10* and *CXCL11* in the NiO and ZnO-exposed groups increased transiently at three days or one week or one month following intratracheal instillation, and no difference was observed in the expression level between nanomaterials of high and low pulmonary toxicity.

### 3.2. Assessment of the Accuracy of Gene Expression of the Toxicity of Chemicals

Table 3 shows the results of the receiver operating characteristics (ROC) for the toxicity of the nanomaterials by the gene expression. Recognizing NiO and CeO_2_ as substances with high pulmonary toxicity and TiO_2_ and ZnO as substances with low pulmonary toxicity, we examined whether or not this pulmonary toxicity ranking was related to the expression of the five chemokine genes. The maximum areas under the curves (AUC) of each gene were generally observed at one week and one month following intratracheal instillation. There was a considerable relationship between the pulmonary toxicity of the nanomaterials and the gene expression of *CXCL5*, *CCL2*, and *CCL7*, with AUC of 0.95 or higher at one week and one month. Further, false negatives could be minimized when evaluated with the combination of *CXCL5*, *CCL2*, and *CCL7* (Table 4). On the other hand, there seemed to be a relatively low relationship between the pulmonary toxicity of the nanomaterials and gene expressions of *CXCL10* and *CXCL11* during the other observation times.

### 3.3. CXCL5, CCL2 and CCL7 Immunostaining

**Figure 2:**
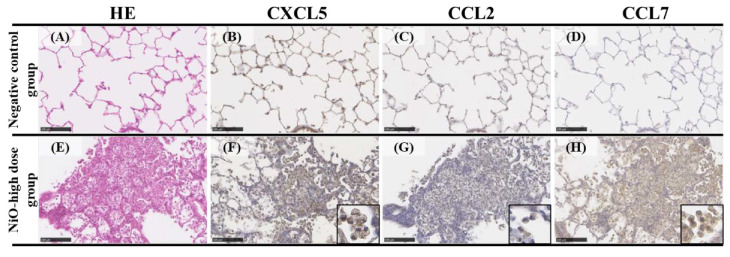
Representative images of CXCL5, CCL2, and CCL7 immunostaining in lung tissue exposed to NiO. (**A**–**D**): the negative control lungs; (**A**) H&E staining; (**B**) CXCL5 immunostaining; (**C**) CCL2 immunostaining; (D)CCL7 immunostaining. (**E**–**H**): the NiO-high dose exposed lungs; (**E**) H&E staining; (**F**) CXCL5 immunostaining; (**G**) CCL2 immunostaining; (**H**) CCL7 immunostaining. All of the examples illustrate findings at 1 month after intratracheal instillation: Positive cells of CXCL5, CCL2, and CCL7 immunostaining on NiO-exposed lungs were mainly macrophages. (internal scale bar = 100 μm for all).

Figure 2 shows CXCL5, CCL2, and CCL7 immunostaining in the NiO-high dose group and the negative control group at one month after intratracheal instillation. The CXCL5 immunostaining positive cells were mainly observed at the gathering sites of inflammatory cells, mainly macrophages, at one month in the high dose of NiO exposure group. Similar to CXCL5, the positive cells of CCL2 and CCL7 immunostaining were observed in aggregations of inflammatory cells centered on macrophages.

### 3.4. Pathological Features in the Rat Lungs

**Figure 3:**
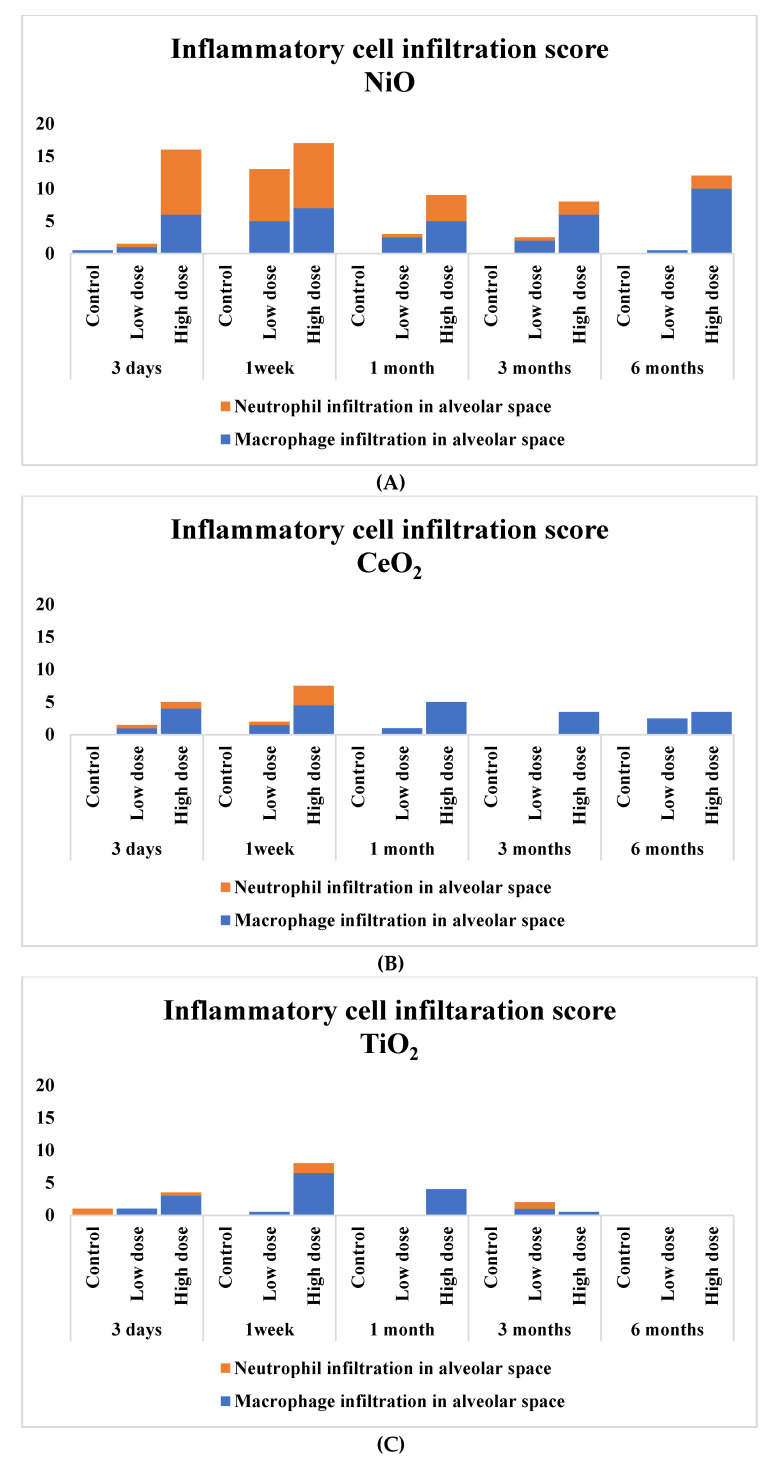

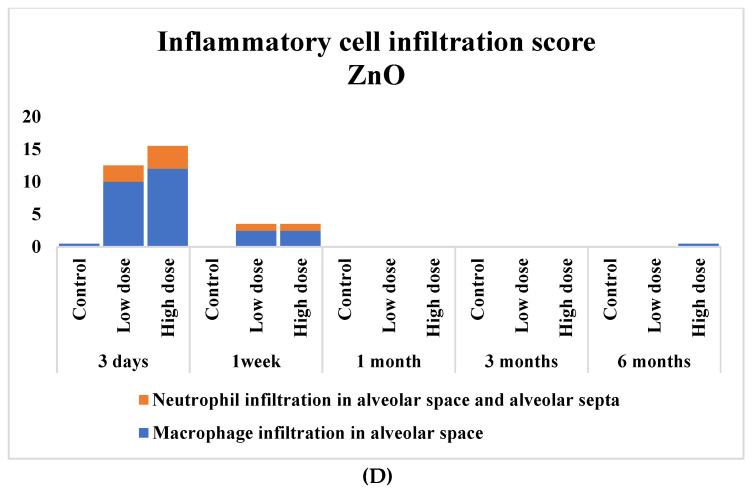
Score of inflammatory cell infiltration in the lung exposed to nanomaterials. (**A**) the NiO-exposed group; (**B**) the CeO_2_-exposed group; (**C**) the TiO_2_-exposed group; (**D**)the ZnO exposed-group. The severity of lung histological changes in the negative control and nanoparticle-exposed rats was scored as none (0), minimal (0.5), mild (1), moderate (2), or severe (3). Exposure to NiO and CeO_2_ resulted in persistent infiltration of inflammatory cells throughout the observation period, but TiO_2_ and ZnO only showed a transient infiltration. The score is calculated with a following equation. Σ (grade × number of animals with grade). The low dose groups and the high dose groups were exposed 0.2 mg 1.0 mg, respectively.

**Figure 4:**
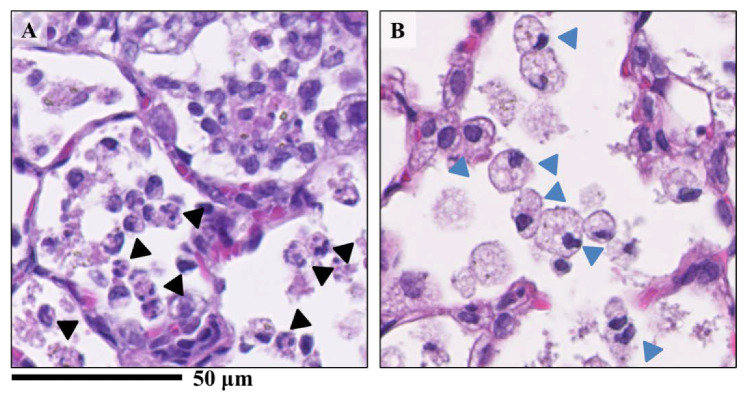
Lung samples sectioned with H&E staining exposed to nanomaterials intratracheally. (**A**) the NiO-high dose exposed group; (**B**) the CeO_2_-high dose group. There were differences of infiltrating inflammatory cells between nanomaterials. While mainly neutrophils and macrophages were found in the alveoli in the NiO-high dose group (**A**), macrophage-based inflammatory cell infiltration was observed in the CeO_2_-high dose group (**B**). Black arrow heads indicate neutrophils and blue arrow heads indicates macrophages.

**Figure 5:**
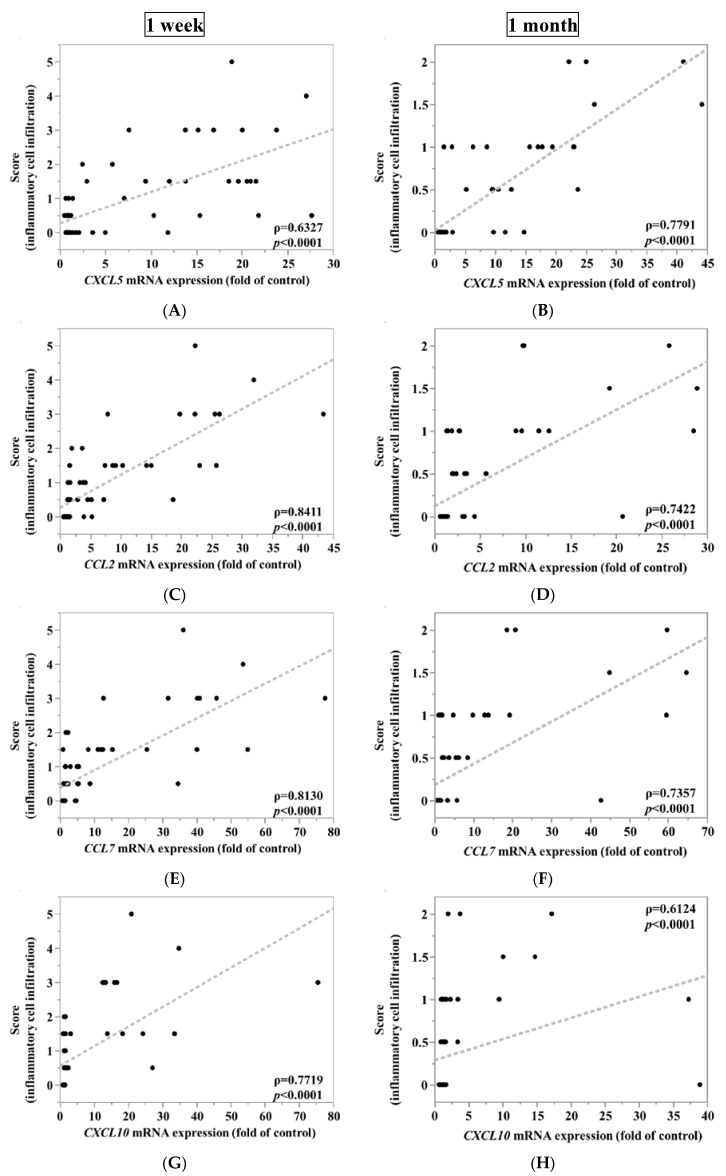

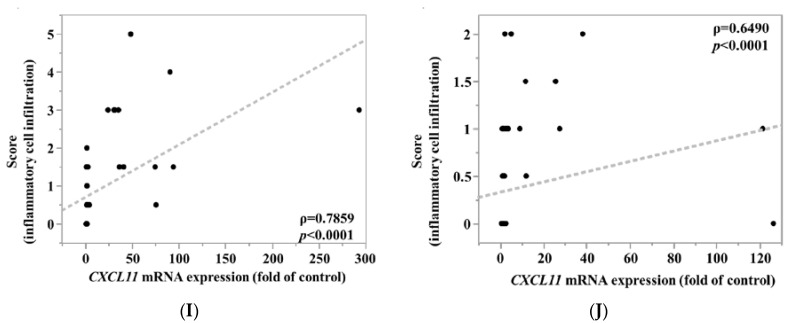
Relationship between inflammatory cell infiltration and gene expression of each 5 chemokines in exposed lung. (**A**) *CXCL5* mRNA expression, (**C**) *CCL2* mRNA expression, (**E**) *CCL7* mRNA expression, (**G**) *CXCL10* mRNA expression, (**I**) *CXCL11* mRNA expression at 1 week after the instillation versus score of inflammatory cell infiltration and (**B**) *CXCL5* mRNA expression, (**D**) *CCL2* mRNA expression, (**F**) *CCL7* mRNA expression, (**H**) *CXCL10* mRNA expression, (**J**) *CXCL11* mRNA expression at 1 month after the instillation versus score of inflammatory cell infiltration. There was relatively good correlation between inflammatory cell infiltration in lung tissues and *CXCL5*, *CCL2*, and *CCL7* at one week and one month after intratracheal instillation.

Figure 3 shows the inflammatory cell infiltration scores of the pathological features in the rat lungs. There was sustained inflammation in the rat lungs that were exposed to NiO and CeO_2_, while only transient inflammation was observed in rat lungs exposed to TiO_2_ and ZnO. In Figure 4, pulmonary inflammation appears mainly in the macrophages and neutrophils in the rat lungs that were exposed to NiO, and in the macrophages in the rat lungs exposed to CeO_2_, respectively. We previously reported that inflammatory cells influx in the bronchoalvelar lavage fluid was persistent in the NiO and CeO_2_ groups, whereas it was transient in the TiO_2_ and ZnO groups and these findings were similar to the pathological features of each group [19,20,21]. Figure 5 shows the relationship between the inflammatory cell score and the gene expression of *CXCL5*, *CCL2*, *CCL7*, *CXCL10,* and *CXCL11* in the lung exposed to the nanomaterials. The expression levels of *CXCL5*, *CCL2*, and *CCL7* at one week and one month correlated moderately or strongly with the degree of inflammatory cell infiltration in the lung tissue.

## 4. Discussion

According to the microarray analysis in our experiments, the inflammation-related genes that showed an upregulation in the lungs of rats injected intratracheally with NiO nanoparticles were *CXCL5*, *CCL2*, *CCL7*, *CXCL10*, and *CXCL11*, all of which are chemokine genes. All of these chemokines are involved in inflammatory responses through the migration, accumulation, and activation of inflammatory cells, such as neutrophils and macrophages [33,34,35,36].

CXCL5 is a CXC chemokine with a glutamate-leucine-arginine (ELR) motif (ELR + chemokine) and it has potent chemotactic and activating functions of neutrophils in the lung [37]. In our experiments, the gene expression of *CXCL5* was persistently increased by NiO and CeO_2_, which have high pulmonary toxicity, and not, or only transiently, increased by TiO_2_ and ZnO, which have low pulmonary toxicity (Figure 1). In other studies, the expression of *CXCL5* by respirable chemicals was enhanced in the lungs of rodents exposed to single-wall carbon nanotubes and inflammogenetic stainless-steel welding fumes [38,39]. For lung lesion other than pulmonary inflammation, exposure to cigarette smoke, which leads to lung cancer and COPD, also induced *CXCL5* expression in the lung [40]. Alternatively, no up-regulation of *CXCL5* was reported in rat lungs that were exposed to C60 fullerenes with low toxicity [41].

The chemokines CCL2 and CCL7 are known as chemotactic agents for monocytes and they have been found to play a key role in mediating lung inflammation. It is also known that CCL2 and CCL7 are both CC chemokines, are closely related to each other, have a common receptor (CCR2), and elicit similar responses, such as those that are involved in the migration of macrophages, lymphocytes, and neutrophils [42]. Similar to *CXCL5* in our experiment, exposure to NiO and CeO_2_ showed a continuous, increasing trend of the expression of *CCL2* and *CCL7*, but exposure to TiO_2_ and ZnO did so only transiently (Figure 1). Langley et al. found that *CCL2* and *CCL7* were persistently upregulated in rat lungs that were exposed to inhaled crystalline silica, a dust with high toxicity [43]. Fujita et al. also reported that *CCL2* and *CCL7* were upregulated in the lungs of rats injected intratracheally with inflammogenetic single-wall carbon nanotube (SWCNT) [38,44]. Abdelgied et al. found that *CCL2* expression was increased in rat lungs following intratracheal instillation of potassium octatitanate fibers (POT fibers), which are suggested to have carcinogenic potential [45]. On the other hand, it has also been reported that *CCL2* and *CCL7* expression was transient in rat lungs following the intratracheal instillation of C60 fullerenes with low toxicity [41].

The expression of *CXCL5*, *CCL2*, and *CCL7* had a certain level of correlation with the lung inflammation score in our experiment, and these findings suggested that these gene expressions were involved in lung inflammation as the main pathological lesion from exposure to nanomaterials. Immunostaining results revealed that CXCL5, CCL2, and CCL7 mainly stained macrophages in the infiltration of inflammatory cells in the alveolar space, although only lung tissues that were exposed to NiO were examined. This suggests that these chemokines in the alveolar space also contributed to the formation of pulmonary inflammation.

CXCL10 and CXCL11 are ELR- CXC chemokines that have CXCR3 as a common receptor [46]. No difference in the expression level between nanomaterials of high and low pulmonary toxicity was observed in our experiment. Fundamentally, CXCL10 and CXCL11, which are Th1 network-related cytokines, seem to have little relation to this pathological condition in which the inflammatory cells are mainly macrophages and neutrophils. The T cells were rarely involved in the lesions in the present experiment, which is the reason why these genes expressions are not reflected in the ranking.

There was a considerable relationship between the toxicity ranking of the nanomaterials and the expression of *CXCL5*, *CCL2* and *CCL7*, which have AUC with high values. Under the condition of high AUC, low doses of highly toxic substances produced more gene expression than high doses of less toxic substances. The dosage settings in this experiment, 0.8 mg/kg BW and 4 mg/kg BW, were necessary for considering the validity of the expression level of the three genes. We considered that the low and high dosages were approximately the minimum and maximum doses that are necessary for evaluating the pulmonary toxicity of the metal oxide nanoparticles in our experiment. The low dose is approximately the minimum dose at which nanomaterials with high toxicity induced pulmonary inflammation. We previously injected NiO at 0.8 mg/kg BW in rats of a different species than those in the present experiment, and there was similar mild neutrophil inflammation [10]. The dose of 4 mg/kg BW was considered to be the maximum dose that did not cause overload in intratracheal instillation studies of nanoparticles. We previously reported that doses in excess of 4 mg/kg induced pulmonary surplus inflammation and the delay of the biological half time of nanoparticles [47]. Morrow PE et al. and Bellmann B et al. reported in toner studies, as well, which a delayed clearance of alveolar macrophages occurred between 1 mg/rat (4 mg/kg BW in our experiment is equivalent to 1 mg/rat) and 3 mg/rat of lung deposition [48,49], indicating that the threshold of overload is between 1 and 3 mg/rat. It was speculated from these data that exposure to doses above 1 mg/rat might induce pulmonary toxicity by the chemicals themselves as well as toxicity from the excessive dose. These doses, 0.8 mg/kg BW and 4 mg/kg BW, as the burden on the lungs of nanomaterials after intratracheal instillation, may correspond to approximately 0.36 and 1.8 years of the inhalation period at a concentration of 3 mg/m^3^, respectively, as the maximum concentration for humans of inhalable dust without crystalline silica (working time 8 h/day, five days/week) defined by the American Conference of Governmental Industrial Hygienists (ACGIH).

As for the examination of the observation times, there were high AUCs between the pulmonary toxicity of the nanomaterials and the gene expression of the three chemokines at one month and one week following intratracheal instillation. In the acute phases, for example, at around three days after intratracheal instillation, there may have been a bolus effect in which even low-toxic substances induced pulmonary inflammation. Yoshiura et al. reported that even TiO_2_ (P90), which did not cause pulmonary tumors in two years observation time following intratracheal instillation, induced transient inflammation at three days in the same intratracheal instillation study [27]. ZnO is known to be a soluble metal oxide that causes inflammation, and in our experiment, ZnO nanoparticles, among the four nanomaterials studied, induced severe pulmonary inflammation at three days following intratracheal instillation. At one month, the pulmonary inflammation by the bolus effect had disappeared, and the gene may have been expressed due to the toxicity of the original nanomaterial. Although some inflammation remained in the low toxic substance exposure group at one week (Figure 3), there was a difference in the degree of gene expression between the high and low toxic nanomaterials. It is considered that the decrease in the expression of chemokines may have proceeded from the cessation of inflammation at one week following intratracheal instillation. Oh JH et al. reported that the expression of *CXCL5*, *CCL2,* and *CCL7* in rat lungs from inhaled stainless-steel welding fumes returned to negative control levels earlier than inflammation as pathological changes in the lung [39].

Regarding the up-regulation of these three genes, there seems to be no common transcription factor, but it may be involved in the activation of TGF-β-activated kinase 1 (TAK1), which activates NF-κB, JNK, and p38 MARK, which are known transcription factors and regulatory factors for many chemokines. Thiesfes et al. reported that the gene expression of *CXCL5*, *CCL2*, and *CCL7* in the same experimental condition was stimulated by TNF through TAK1 activity in NIH3T3 cells [50]. Li et al. also reported that TAK1 inhibition suppressed inflammation and fibrosis in a pneumoconiosis animal model [51].

As described above, because the gene expression of the three chemokines is stable and reflects the difference in pulmonary toxicity of nanomaterials between different doses (0.8 and 4 mg/kg BW), and different observation periods (one week and one month as observation times), these three chemokine genes, *CXCL5*, *CCL2*, and *CCL7*, are considered to be useful as biomarkers for the ranking of the pulmonary toxicity of nanomaterials.

When the pulmonary toxicity of the nanomaterials was screened in this intratracheal instillation model, it is important that there were no false negatives. If the screening criteria for pulmonary toxicity of nanomaterials is that any of the three gene expressions exceed the cut-off value, false negatives as a screening test would have been minimum in this experiment (Table 4). Therefore, we think that the combination of these three genes, *CXCL5*, *CCL2*, and *CCL7*, is useful for screening the pulmonary toxicity of nanomaterials.

## 5. Conclusions

We analyzed the mRNA expression of chemokines in rat lungs following intratracheal instillation of four different nanomaterials in order to find useful predictive markers of the pulmonary toxicity of nanomaterials. Our results suggest that three chemokine genes, especially *CXCL5*, followed by *CCL2* and *CCL7*, can be useful as biomarkers for the ranking of the pulmonary toxicity of nanomaterials.

## Figures and Tables

**Table 1:** Physiochemical characterization of the nanomaterials used in the present study.

Nanomaterials	NiO	CeO_2_	TiO_2_	ZnO
Pulmonary toxicity	High	High	Low	Low
Primary diameter	19 nm	7.8 nm	Short 12 nm Long 55 nm	35 nm
Specific surface area	57 m^2^/g	101 m^2^/g	111 m^2^/g	31 m^2^/g
Shape	Sphere	Irregular shape	Spindle-shaped	Sphere like
Secondary diameter (DLS)	39.8–47.1 nm	2.6–9.3 nm	20–80 nm	17–37 nm
Purity	More than 99.5%	99.9%	99.5%	99.9%
Bulk density	6.72 g/cm^3^	7.22 g/cm^3^	4.17 g/cm^3^	5.60 g/cm^3^
Solubility	Low (>CeO_2_)	Low	Low	High

Primary particle diameter: the particle diameter of the one particle; Secondary particle diameter: the particle diameter of agglomerate; DLS: Dynamic light scattering.

**Table 2:** (**A**) Number of genes by mRNA expression level in the NiO-high dose group at one month. (**B**) Description of the genes that are related to ‘inflammatory response’ among the 16 genes upregulated ≧ 8-fold.

(A)			
mRNA Level (Fold Change of Control)			Number of Genes
Up regulation	≧2-fold		450
	2–4-fold		383
	4–8-fold		51
	≧8-fold		16
Down regulation	≦12-fold		388
	12–14-fold		359
	14–18-fold		15
	≦18-fold		14
**(B)**			
**Gene Symbol**	**Gene Description**		**Fold Change**
*CXCL5*	Chemokine (C-X-C motif) ligand5		12.83
*CCL2*	Chemokine (C-C motif) ligand2		17.93
*CCL7*	Chemokine (C-C motif) ligand7		23.44
*CXCL10*	Chemokine (C-X-C motif) ligand10		16.42
*CXCL11*	Chemokine (C-X-C motif) ligand11		16.70

Table 2 (A) shows the number of genes by mRNA expression level in the NiO-high dose group at one month after intratracheal instillation among 20,174 genes examined using cDNA microarray. (B) There were five genes related to inflammation of the 16 genes in the analysis using Database for Annotation Visualization and Integrated Discovery 6.8 (DAVID 6.8, https://david.ncifcrf.gov).

**Table 3:** Receiver operating characteristic (ROC) analysis between gene expression and pulmonary toxicity of nanomaterials.

Observation Time	3 Days	1 Week	1 Month	3 Months	6 Months
	AUC	AUC	AUC	AUC	AUC
	(95% C.I.)	(95% C.I.)	(95% C.I.)	(95% C.I.)	(95% C.I.)
	(Cut-off Value)	(Cut-off Value)	(Cut-off Value)	(Cut-off Value)	(Cut-off Value)
*CXCL5*	0.990	0.998	0.995	0.983	0.928
	(0.982–0.999)	(0.961–1.000)	(0.948–1.000)	(0.874–0.998)	(0.803–0.976)
	(10.008)	(10.308)	(9.513)	(3.277)	(1.934))
*CCL2*	0.693	0.973	0.980	0.910	0.848
	(0.501–0.835)	(0.841–0.996)	(0.904–0.996)	(0.753–0.971)	(0.674–0.937)
	(11.495)	(3.919)	(1.945)	(2.059)	(1.076)
*CCL7*	0.680	0.968	0.993	0.898	0.893
	(0.487–0.826)	(0.860–0.993)	(0.933–0.999)	(0.744–0.963)	(0.720–0.964)
	(12.380)	(5.090)	(1.990)	(2.007)	(1.020)
*CXCL10*	0.603	0.795	0.848	0.778	0.663
	(0.415–0.764)	(0.592–0.912)	(0.673–0.974)	(0.593–0.894)	(0.473–0.811)
	(8.311)	(2.088)	(1.620)	(1.466)	(1.097)
*CXCL11*	0.525	0.890	0.883	0.813	0.920
	(0.340–0.704)	(0.744–0.958)	(0.741–0.952)	(0.612–0.922)	(0.798–0.971)
	(7.843)	(2.790)	(2.073)	(1.025)	(0.706)

AUC: Area under the curve; 95% C.I.: 95% Confidence interval.

**Table 4:** Sensitivity and specificity of gene expression of five chemokines in the pulmonary toxicity of nanomaterials.

		Sensitivity		Specificity		False Positive		False Negative	
3 days	*CXCL5*	0.95	(19/20)	1.00	(20/20)	0.00	(0/20)	0.05	(1/20)
	*CCL2*	0.50	(10/20)	0.95	(19/20)	0.05	(1/20)	0.50	(10/20)
	*CCL7*	0.50	(10/20)	0.95	(19/20)	0.05	(1/20)	0.50	(10/20)
	*CXCL10*	0.30	(6/20)	1.00	(20/20)	0.00	(0/20)	0.70	(14/20)
	*CXCL11*	0.35	(7/20)	0.95	(19/20)	0.05	(1/20)	0.65	(13/20)
	*CXCL5 + CCL2 + CCL7*	0.90	(18/20)	0.05	(1/20)	0.95	(19/20)	0.10	(2/20)
1 week	*CXCL5*	0.95	(19/20)	1.00	(20/20)	0.00	(0/20)	0.05	(1/20)
	*CCL2*	1.00	(20/20)	0.90	(18/20)	0.10	(2/20)	0.00	(0/20)
	*CCL7*	0.95	(19/20)	0.90	(18/20)	0.10	(2/20)	0.05	(1/20)
	*CXCL10*	0.75	(15/20)	0.90	(18/20)	0.10	(2/20)	0.25	(5/20)
	*CXCL11*	0.75	(15/20)	0.95	(19/20)	0.05	(1/20)	0.25	(5/20)
	*CXCL5 + CCL2 + CCL7*	1.00	(20/20)	0.90	(18/20)	0.10	(2/20)	0.00	(0/20)
1 month	*CXCL5*	0.95	(19/20)	1.00	(20/20)	0.00	(0/20)	0.05	(1/20)
	*CCL2*	1.00	(20/20)	0.90	(18/20)	0.10	(2/20)	0.00	(0/20)
	*CCL7*	1.00	(20/20)	0.95	(19/20)	0.05	(1/20)	0.00	(0/20)
	*CXCL10*	0.65	(13/20)	1.00	(20/20)	0.00	(0/20)	0.35	(7/20)
	*CXCL11*	0.65	(13/20)	0.95	(19/20)	0.05	(1/20)	0.35	(7/20)
	*CXCL5 + CCL2 + CCL7*	1.00	(20/20)	0.90	(18/20)	0.10	(2/20)	0.00	(0/20)
3 months	*CXCL5*	1.00	(20/20)	0.95	(19/20)	0.05	(1/20)	0.00	(0/20)
	*CCL2*	0.95	(19/20)	0.80	(16/20)	0.20	(4/20)	0.50	(1/20)
	*CCL7*	0.95	(19/20)	0.80	(16/20)	0.20	(4/20)	0.50	(1/20)
	*CXCL10*	0.75	(15/20)	0.80	(16/20)	0.20	(4/20)	0.25	(5/20)
	*CXCL11*	0.90	(18/20)	0.75	(15/20)	0.25	(5/20)	0.10	(2/20)
	*CXCL5 + CCL2 + CCL7*	1.00	(20/20)	0.80	(16/20)	0.20	(4/20)	0.00	(0/20)
6 months	*CXCL5*	0.95	(19/20)	0.80	(16/20)	0.20	(4/20)	0.05	(1/20)
	*CCL2*	0.85	(17/20)	0.80	(16/20)	0.20	(4/20)	0.15	(3/20)
	*CCL7*	0.85	(17/20)	0.85	(17/20)	0.15	(3/20)	0.15	(3/20)
	*CXCL10*	0.55	(11/20)	0.80	(16/20)	0.20	(4/20)	0.45	(9/20)
	*CXCL11*	1.00	(20/20)	0.70	(14/20)	0.30	(6/20)	0.00	(0/20)
	*CXCL5 + CCL2 + CCL7*	1.00	(20/20)	0.30	(6/20)	0.70	(14/20)	0.00	(0/20)

Sensitivity = (the number of true positives/the number of true positives + the number of false negatives). Specificity = (the number of true negatives/the number of false positives + the number of true negatives). False positive = (the number of false positives/the number of false positives + the number of true negatives). False negatives = (the number of false negatives/the number of true positives + the number of false negatives).

## Appendix A

**Table A1:** (**A**) Confusion matrix. (**B**) Example of confusion matrix.

(**A**) Confusion matrix			
		**Pulmonary Toxicity**	
		**High**	**Low**
Gene expression level (fold of control)	≧Cut off value	a (True positives)	b (False positives)
	<Cut off value	c (False negatives)	d (True negatives)
		a + c	b + d
Sensitivity = $\frac{\mathrm{The}\;\mathrm{number}\;\mathrm{of}\;\mathrm{true}\;\mathrm{positives}}{\mathrm{The}\;\mathrm{number}\;\mathrm{of}\;\mathrm{true}\;\mathrm{positives}\;+\;\mathrm{The}\;\mathrm{number}\;\mathrm{of}\;\mathrm{false}\;\mathrm{negatives}\;}$ = $\frac{a}{a+c}$ Specificity = $\frac{\mathrm{The}\;\mathrm{number}\;\mathrm{of}\;\mathrm{true}\;\mathrm{negatives}}{\mathrm{The}\;\mathrm{number}\;\mathrm{of}\;\mathrm{false}\;\mathrm{positives}\;+\;\mathrm{The}\;\mathrm{number}\;\mathrm{of}\;\mathrm{true}\;\mathrm{negatives}\;}$ = $\frac{d}{b+d}$ False positive = $\frac{\mathrm{The}\;\mathrm{number}\;\mathrm{of}\;\mathrm{false}\;\mathrm{positives}}{\mathrm{The}\;\mathrm{number}\;\mathrm{of}\;\mathrm{false}\;\mathrm{positives}\;+\;\mathrm{The}\;\mathrm{number}\;\mathrm{of}\;\mathrm{true}\;\mathrm{negatives}\;}$ = $\frac{b}{b+d}$ False negative = $\frac{\mathrm{The}\;\mathrm{number}\;\mathrm{of}\;\mathrm{false}\;\mathrm{negatives}}{\mathrm{The}\;\mathrm{number}\;\mathrm{of}\;\mathrm{true}\;\mathrm{positives}+\;\mathrm{The}\;\mathrm{number}\;\mathrm{of}\;\mathrm{false}\;\mathrm{negatives}\;}$ = $\frac{c}{a+c}$ a: The number of true positives, b: The number of false positives. c: The number of false negatives, d: The number of true negatives.			
(**B**) Example of confusion matrix.			
	***CXCL5*** **at 1 week**	**Pulmonary Toxicity**	
		**High**	**Low**
Gene expression level (fold of control)	≧Cut off value	19	0
	<Cut off value	1	20
		20	20
For example, in *CXCL5* at 1 week after instillation. Sensitivity = $\frac{19}{19\;+\;1}$ = 0.95, Specificity = $\frac{20}{0\;+\;20}$ = 1.00, False positive = $\frac{0}{0\;+\;20}$ = 0.00, False negative = $\frac{1}{19\;+\;1}$ = 0.05.			

**Table A2:** *p* values of gene expression of 5 chemokines in lung exposed to nanomaterials.

***CXCL5***	**3 Days**	**1 Week**	**1 Month**	**3 Months**	**6 Months**
**Low Dose Group**					
NiO	0.0037 **	0.0008 **	0.0134 *	0.0056 **	0.1862
CeO_2_	0.0002 **	<0.0001 **	0.0003 **	0.0302 **	0.3518
TiO_2_	0.4254	0.4474	0.9	0.2214	0.8551
ZnO	0.1483	0.6346	0.7478	0.3446	0.1679
***CCL2***	**3 Days**	**1 Week**	**1 Month**	**3 Months**	**6 Months**
**Low Dose Group**					
NiO	0.5879	0.0017 **	0.8141	0.8456	0.4627
CeO_2_	0.0170 *	0.1357	0.2553	0.5301	0.9883
TiO_2_	0.9217	0.9928	0.737	0.1995	0.5812
ZnO	0.0011 **	0.926	0.6449	0.358	0.2674
***CCL7***	**3 Days**	**1 Week**	**1 Month**	**3 Months**	**6 Months**
**Low Dose Group**					
NiO	0.8425	0.0018 **	0.8701	0.9175	0.608
CeO_2_	0.1495	0.3895	0.4254	0.8892	1
TiO_2_	0.9424	0.9997	0.9087	0.2562	0.6004
ZnO	0.0019 **	0.5976	0.6198	0.3636	0.1821
***CXCL10***	**3 Days**	**1 Week**	**1 Month**	**3 Months**	**6 Months**
**Low Dose Group**					
NiO	0.9877	0.0053 **	0.9959	0.8678	0.3692
CeO_2_	0.161	0.4941	0.5374	0.9784	0.3855
TiO_2_	0.9956	0.9929	0.7097	0.212	0.592
ZnO	<0.0001 **	0.0002 **	0.4146	0.3867	0.3168
***CXCL11***	**3 Days**	**1 Week**	**1 Month**	**3 Months**	**6 Months**
**Low Dose Group**					
NiO	0.9995	0.0289 *	0.9985	0.8235	0.4274
CeO_2_	0.2719	0.5391	0.5132	0.9954	0.6872
TiO_2_	0.9996	0.9982	0.7218	0.2127	0.2207
ZnO	<0.0001 **	0.0038 **	0.7349	0.3887	0.1187
***CXCL5***	**3 Days**	**1 Week**	**1 Month**	**3 Months**	**6 Months**
**High dose group**					
NiO	0.0063 **	0.0003 **	<0.0001 **	<0.0001 **	<0.0001 **
CeO_2_	0.0004 **	<0.0001 **	<0.0001 **	<0.0301 *	0.0020 **
TiO_2_	<0.0001 **	0.0053 **	0.0133 *	0.7013	0.4984
ZnO	0.1626	0.6737	0.6125	0.4968	0.0405 *
***CCL2***	**3 Days**	**1 Week**	**1 Month**	**3 Months**	**6 Months**
**High Dose Group**					
NiO	<0.0001 **	0.0022 **	0.0003 **	0.0025 **	<0.0001 **
CeO_2_	<0.0001 **	0.0156 *	0.0130 *	0.0004 **	0.0177 *
TiO_2_	<0.0001 **	0.1091	0.0267 *	0.8836	0.9128
ZnO	0.0020 **	0.6238	0.9559	0.4076	0.3763
***CCL7***	**3 Days**	**1 Week**	**1 Month**	**3 Months**	**6 Months**
**High Dose Group**					
NiO	<0.0001 **	0.0031 **	0.0004 **	0.0064 **	<0.0001 **
CeO_2_	<0.0001 **	0.0889	0.0782	0.0141 *	0.0327 *
TiO_2_	0.0008 **	0.2082	0.0651	0.9479	0.8681
ZnO	0.0110 *	0.5797	0.9974	0.4146	0.8918
***CXCL10***	**3 Days**	**1 Week**	**1 Month**	**3 Months**	**6 Months**
**High Dose Group**					
NiO	0.0004 **	0.2108	0.0081 **	0.1018	0.9967
CeO_2_	0.3798	0.5606	0.4095	0.1205	0.5615
TiO_2_	0.0613	0.2908	0.9398	0.9278	0.3888
ZnO	0.0011 **	0.0655	0.54	0.4204	0.659
***CXCL11***	**3 Days**	**1 Week**	**1 Month**	**3 Months**	**6 Months**
**High Dose Group**					
NiO	0.0014 **	0.6045	0.0277 *	0.097	0.2332
CeO_2_	0.2058	0.5708	0.4182	0.1515	0.2836
TiO_2_	0.1343	0.3348	0.7182	0.9359	0.2013
ZnO	<0.0001 **	0.0672	0.4405	0.3996	0.4958

Asterisks indicate significant differences compared with each control (Dunnett’ test) (* *p* < 0.05, ** *p* < 0.01).
